# Formulation in DDA-MPLA-TDB Liposome Enhances the Immunogenicity and Protective Efficacy of a DNA Vaccine against *Mycobacterium tuberculosis* Infection

**DOI:** 10.3389/fimmu.2018.00310

**Published:** 2018-02-27

**Authors:** Maopeng Tian, Zijie Zhou, Songwei Tan, Xionglin Fan, Longmeng Li, Nadeem Ullah

**Affiliations:** ^1^Department of Pathogen Biology, School of Basic Medicine, Tongji Medical College, Huazhong University of Science and Technology, Wuhan, China; ^2^Tongji School of Pharmacy, National Engineering Research Center for Nanomedicine, Huazhong University of Science and Technology, Wuhan, China

**Keywords:** dimethyldioctadecylammonium, trehalose 6,6′-dibehenate, monophosphoryl lipid A, liposome, adjuvant, DNA vaccine, tuberculosis

## Abstract

Despite the vaccine *Mycobacterium bovis* Bacillus Calmette–Guérin is used worldwide, tuberculosis (TB) remains the first killer among infectious diseases. An effective vaccine is urgently required. DNA vaccine has shown prophylactic as well as therapeutic effects against TB, while its weak immunogenicity hinders the application. As a strong inducer of Th1-biased immune response, DMT, consisting of dimethyldioctadecylammonium (DDA) and two pattern recognition receptor agonists monophosphoryl lipid A and trehalose 6,6′-dibehenate (TDB), was a newly developed liposomal adjuvant. To elucidate the action mechanism of DMT and improve immunological effects induced by DNA vaccine, a new recombinant eukaryotic expression plasmid pCMFO that secretes the fusion of four multistage antigens (Rv2875, Rv3044, Rv2073c, and Rv0577) of *Mycobacterium tuberculosis* was constructed. pCMFO/DDA and pCMFO/DMT complexes were then prepared and their physicochemical properties were analyzed. The immunogenicity and protection against *M. tuberculosis* infection in vaccinated C57BL/6 mice were compared. Formulation of DNA and two agonists into the DDA liposome decreased zeta potential but increased the stability of storage, which resulted in a slower and longer-lasting release of DNA from the DNA–DMT complex than the DNA–DDA liposome. Besides Th1-biased responses, pCMFO/DMT vaccinated mice elicited more significantly CFMO-specific IL2^+^ T_CM_ cell responses in the spleen and provided an enhanced and persistent protection against *M. tuberculosis* aerosol infection, compared to pCMFO/DDA and pCMFO groups. Therefore, the adjuvant DMT can release DNA and agonists slowly, which might attribute to the improved protection of DMT adjuvanted vaccines. pCMFO/DMT, a very promising TB vaccine, warrants for further preclinical and clinical trials.

## Introduction

Tuberculosis (TB) remains the first killer among infectious diseases worldwide, despite *Mycobacterium bovis* Bacillus Calmette–Guérin (BCG), the only available attenuated live vaccine for TB, has been integrated into Expanded Program of Immunization since 1974 (1). Moreover, the increasing incidence of coinfection with human immunodeficiency virus (HIV) and the emergence of multidrug-resistant-TB and extensively drug-resistant-TB deteriorate the threat of TB to the public health (1). Therefore, an effective prophylactic as well as therapeutic vaccine is urgently required.

Tuberculosis vaccine candidates generally fall into two categories. One belongs to recombinant live vaccines such as modified BCG strains, attenuated *Mycobacterium tuberculosis* strains, and recombinant viral vaccines. The other type, namely, subunit vaccines include DNA and protein subunits. Currently, there have been increasing interests to develop subunit vaccines, which were constructed based on the defined components with safety and five candidates are being conducted in the different phases of clinical trials (2). Among all types, DNA vaccine is the easiest for mass production. In particular, DNA vaccine shows both prophylactic (3, 4) and therapeutic (5) effects against TB. Traditionally, the choice of genes has been thought as the most important factor that influences the efficacy of DNA vaccine (6). *Mycobacterium leprae Hsp65* and *Ag85A* of *M. tuberculosis* (3, 4) were initially used for the development of DNA vaccine against TB. Subsequently, at least 60 genes from the genome of *M. tuberculosis* were constructed as DNA vaccine candidates, which could evoke antigen-specific humoral and cell-mediated immune responses and confer various degrees of protection against virulent *M. tuberculosis* infection in mice (6). However, weak immunogenicity of DNA vaccines in larger mammals significantly hinders the practical use, as evidenced by a few DNA vaccines against HIV (7, 8), HBV (9), and malaria (10). To this end, the combination of multiple monovalent DNAs (11) and the fusion DNA of multiple genes (12, 13) have been designed to improve the protection. Meanwhile, immunomodulators (14) and various delivery methods such as gene gun (15), electroporation (16), as well as administration with chitosan (17), PLGA (18), or liposome (19) also have been developed to enhance the efficacy of DNA vaccine. However, no DNA vaccines based on these strategies can provide the comparable protection against *M. tuberculosis* infection in mice, as BCG does. The strategy for developing an effective DNA vaccine against TB remains to be improved.

Recently, we reported that both actively growing and latent *M. tuberculosis* strains coexist *in vivo* in active TB patients and latent TB infections (20–22). When encountering transformational environments during infection, *M. tuberculosis* expresses different immunodominant antigens to escape the pre-established immunity in a presumptive infected host (23). Antigen Rv0577 is secreted by actively replicating bacteria (24), while antigens Rv2875, Rv3044, and Rv2073c are characteristically expressed by *M. tuberculosis* in hypoxic environments *in vivo* (25). Each antigen alone vaccinated mice only conferred lower protection than BCG (26), although BCG vaccine does not express Rv2073c (27) and also has a lower expression of both Rv2875 (28) and Rv0577 (29) than *M. tuberculosis*. However, these antigens were chosen to construct a fusion protein CMFO, because they were more highly recognized by T cells isolated from both latent TB infections and active TB patients than non-infected healthy persons using whole blood IFN-γ release assay (22). Moreover, the antigen CMFO was emulsified into a novel liposome DMT, consisting of dimethyldioctadecylammonium (DDA), and two pattern recognition receptor agonists monophosphoryl lipid A (MPLA) and trehalose 6,6′-dibehenate (TDB), not only provided the comparative protection against primary infection with *M. tuberculosis* in vaccinated mice as BCG did, but also boosted BCG primed mice to prevent against latent infection and thwart the reactivation (22). In order to explore the action mechanism of the DMT liposome and develop a more effective DNA vaccine, a new DNA vaccine expressing the fusion protein CMFO, called pCMFO, was firstly constructed. The plasmid pCMFO was emulsified with DDA and DMT to form DNA–liposome complexes, respectively. Different physical and chemical properties of both DNA–liposome complexes were analyzed. Their immunogenicity and protective efficacy against *M. tuberculosis* infection were also compared and evaluated with BCG vaccinated C57BL/6 mice.

## Materials and Methods

### Construction of Recombinant Eukaryotic Expression Plasmid pCMFO

The DNA sequence of CMFO was consisted of the tandem-fusion genes encoding the signal peptide of human tissue plasminogen activator (SP_tPA_) and CMFO, which was amplified by polymerase chain reaction (PCR) with primers (F: 5′-CCCAAGCTTGCCACCATGGATGCAATGAAGA-3′, R: 5′-CGGGATCCTTATCGCGGCATCCTGCGCCAGACGAAC-3′) from commercially synthesized plasmid pDC316-CMFO (Life Invitrogen of Shanghai, Shanghai, China). For amplification, the Phusion high-fidelity DNA polymerase (Thermo Fisher Scientific, Inc., MA, USA) was used. The PCR reaction was started with initial denaturation at 98°C for 30 s, and then 98°C for 10 s, 68°C for 20 s, and 72°C for 90 s, 30 cycles followed by one final extension at 72°C for 10 min. PCR products were first digested with restriction enzymes both *Hind*III and *Bam*HI and then inserted into the corresponding sites of the eukaryotic expression vector pVAX1 (Invitrogen, CA, USA). The resultant recombinant plasmid was identified by enzyme digestion and the inserted fragment was further verified by DNA sequencing. The positive recombinant plasmid named pCMFO was amplified by cultivating the plasmid-transformed *Escherichia coli* (*E. coli*) strain DH5α. Endotoxin-free plasmids were obtained using EndoFree Plasmid Giga kits (Qiagen, CA, USA). The purified plasmids were dissolved in sterile Tris-buffer (10 mM, pH 7.4), aliquoted, and stored at −20°C. The concentration of the plasmid DNA was determined by an Epoch microplate spectrophotometer (BioTek, Vermont, USA).

To confirm the secretory expression of the protein CMFO, 1 × 10^6^ HEK 293T cells (ATCC^®^ CRL-3216™, VA, USA) were seeded in each well of six-well plate and cultured at 5% CO_2_, 37°C in Dulbecco’s modified Eagle medium (Hyclone, UT, USA), supplemented with 10% fetal bovine serum (Hyclone, UT, USA) and 100 U/mL of penicillin and streptomycin. Twenty-four hours later, cells were transfected with the plasmid pCMFO using Lipofectamine 2000 reagent (Invitrogen, CA, USA). The pVAX1-transfected cells were used as negative control. Forty-eight hours after transfection, cells and supernatant were separately collected after low-speed centrifugation at room temperature. The protein CMFO in either cell lysate or supernatant was confirmed by western blotting using mouse anti-CMFO sera (diluted 1/2,000) as the primary antibody (22) and peroxidase-conjugated goat anti-mouse IgG (diluted 1/5,000; Proteintech Biotech, Wuhan, China) as the secondary antibody. Immunoblots were visualized using BeyoECL Plus reagent (Beyotime, Shanghai, China).

### Preparation of Liposomes

Each 100 µL of the DDA liposome (250 µg DDA) and the DMT liposome (250 µg DDA, 25 µg MPLA, and 50 µg TDB) were prepared by lipid film hydration method as previously described (20, 22, 30). Briefly, a batch of 7,500 mg DDA (Sigma-Aldrich, MO, USA) alone, or with 750 mg MPLA (Avanti Polar Lipids Inc., AL, USA) and 1,500 mg TDB (Avanti Polar Lipids Inc., AL, USA), were firstly dissolved in 10 mL chloroform/methanol (9:1, by volume) and then evaporated by blowing N_2_. Sample was maintained overnight under low-pressure condition to form a membrane. Then, liposome was rehydrated in 10 mM sterile Tris-buffer (pH 7.4) at 60°C for 60 min and vortexed every 10 min. pCMFO/DDA and pCMFO/DMT were prepared by emulsifying 100 µL of corresponding liposome with 100 µL of the plasmid pCMFO solution (50 μg/100 μL).

### Characterization of Liposomes

Dynamic light scattering method was used to detect particle size and zeta potential of different liposomes by a ZetaPlus machine (Brookhaven Instruments, New York, NY, USA). The morphology of liposomes was detected by transmission electron microscopy (TEM, Hitachi, Tokyo, Japan). Samples were stained with phosphotungstic acid before observation. To assess the stability, liposomal formulations were kept at 4°C for different days (0, 10, 20, 30, and 60 days). Particle size and zeta potential were further detected. The results are expressed as mean ± SD from three batches.

### DNA Adsorption and Release from DNA–Liposome Complexes

To determine adsorption ability of plasmid DNA to liposome, three batches of DNA–liposome complexes were first equilibrated for 10 min and centrifuged by ultracentrifugation (Beckman Coulter, IL, USA) at 100,000 *g* for 60 min at 4°C. The supernatant was collected and the concentration of DNA in the supernatant was measured. The adsorbed amount of the DNA to the liposome were subtracted the amount of DNA remained in the supernatant from the amount of DNA initially added to the liposome dispersion. The calculated results are expressed as mean ± SD from three batches.

In order to measure the release of the DNA from DNA–liposome complexes, the formulation was first incubated in sterile eppendorf tubes at 37°C. The concentration of DNA in supernatant was determined after centrifugation at appropriate time intervals (every 7 days up to 28 days). The percentage of DNA released from the complex was calculated by the ratio of released DNA in supernatant to the total DNA absorbed into the liposome. The results are expressed as mean ± SD from three batches.

### Mice and Immunization

Specific-pathogen-free female C57BL/6 mice of 6 to 8 weeks age were obtained from the Center for Animal Experiment of Wuhan University (Wuhan, China) and maintained with animal feeding cabinet (VentiRack, CA, USA) in an ABSL-3 biosafety laboratory. Mice were randomly divided into different groups. 200 µL of pCMFO, pCMFO/DDA, or pCMFO/DMT was immunized intramuscularly (i.m.) twice at 3-week intervals (11, 12, 14). 1 × 10^6^ CFU of BCG China were vaccinated subcutaneously (s.c.) once at the time of the first vaccination and used as a positive control (11, 12, 14). PBS, DDA, and DMT alone were used as negative controls. 9 and/or 18 weeks after the first immunization, 6 mice in each group were sacrificed for immunological analysis, and another 6 mice were challenged with virulent *M. tuberculosis* H37Rv strain for the evaluation of protection, respectively. All experiments were repeated twice.

### Challenge with Virulent *M. Tuberculosis* H37Rv Strain

9 and 18 weeks after immunization, different vaccinated mice were challenged with approximately 60 CFU of *M. tuberculosis* H37Rv by an airborne infection apparatus (Glas-col Inc., IN, USA) (30). Four weeks post-challenge, mice in each group (*n* = 6) were sacrificed for the comparison of protective efficacy, which was evaluated by biomarkers including organs bacterial load (log_10_ CFU) and lung pathology as described previously (20–22).

### Antigen-Specific IgG and Subclasses Tittered by ELISA

Sera were collected from each mouse in different vaccinated groups. CMFO, Rv0577, Rv2875, Rv3044, or Rv2073c (22, 31) antigen-specific endpoint titers for IgG, IgG1, and IgG2a (replaced by IgG2c) were detected by ELISA as previously described (21). The results are shown as mean ± SEM log_10_ endpoint titers per group (*n* = 6).

### Cytokines Secreted by Splenocytes Measured by Cytometric Bead Assay or ELISA

Splenocytes were prepared and counted from each mouse in different groups as described previously (32). Subcomponent-specific cytokines Th1 (TNF-α and IFN-γ), Th2 (IL-4), and Th17 (IL-17A) were detected using cytometric bead array (CBA). 5 × 10^6^ cells were plated into each well of a 24-well plate and incubated with RPMI1640 medium (negative control), or RPMI1640 medium with specific proteins including Rv0577, Rv2875, Rv3044, or Rv2073c (each 10 µg/mL), respectively. The supernatant was collected 24 h later and Cytometric Bead Array kit (BD Biosciences, NJ, USA) was performed according to the manufacturer’s instructions.

Alternatively, 5 × 10^6^ cells were incubated with CMFO protein (10 µg/mL) at 37°C and 5% CO_2_. RPMI1640 medium was used as a negative control and PPD (10 mg/mL, Statens Serum Institut, Denmark) was set as a positive control. After an incubation of 72 h, the supernatant was collected and a commercial mouse IFN-γ ELISA kit (Multi Sciences, Hangzhou, China) were used to detect the concentration of IFN-γ (20–22). The results are expressed as mean ± SD (pg/mL) for each group (*n* = 6).

### CMFO-Specific Memory T Cells Analyzed by Intracellular Flow Cytometry

Intracellular flow cytometry analysis was performed as previously described (22). Briefly, 5 × 10^6^ splenocytes were plated in each well of a 24-well plate and incubated with either CMFO (10 µg/mL) or PPD (10 µg/mL) in the presence of 1 µg/mL anti-CD28/CD49d (eBioscience CA, USA). RPMI1640 medium and cell stimulation cocktail (eBioscience, CA, USA) were used as negative and monitoring controls, respectively. Cells were stained with surface markers, including anti-CD4 PE Cy7, anti-CD8a PE, anti-CD44 APC-eFluor^®^ 780, anti-CD62L FITC mAbs, and intracellular markers anti-IFN-γPerCP-Cy5.5 and anti-IL-2 APC mAbs (all from eBioscience, CA, USA). The stained cells were analyzed by an LSRII multicolor flow cytometer (BD Biosciences, CA, USA). The absolute number of CMFO-specific CD4^+^ or CD8^+^ IFN-γ positive T_EM_ (effector memory T cells, CD62L^lo^CD44^hi^) and CD4^+^ or CD8^+^ IL-2 positive T_CM_ (central memory T cells, CD62L^hi^CD44^hi^) cells were analyzed with FlowJo software (Tree Star Inc., OH, USA). The results are represented as mean ± SD per group (*n* = 6).

### Statistical Analysis

Statistical analysis was performed with SPSS Version 19.0 software and differences between multiple groups were performed by single-factor analysis of variance (one-way ANOVA test) with Fisher’s least significant difference posttest. For two-sample comparison, Student’s *t*-test was used. A value of *p* < 0.05 was considered statistically significant.

## Results

### Characterization of Both Liposomes and pCMFO–Liposome Complexes

The recombinant eukaryotic expression plasmid pCMFO was successfully constructed (Figure S1A in Supplementary Material). The protein CFMO was secreted by the plasmid pCMFO-transfected HEK 293T cells, as demonstrated by western blotting (Figure S1B in Supplementary Material). In order to evaluate the effect of liposomal characters on the vaccine-induced protection, physical characters of different liposomes including particle size, polydispersity index (PDI), and zeta potential were characterized as listed in Table 1. Although there was no statistical difference of both particle size and PDI between liposomes DDA and DMT, the incorporation of MPLA and TDB into the DDA liposome resulted in a significant decrease of the surface charge (*p* < 0.05). In addition, the plasmid pCMFO integrated into DDA or DMT to form different DNA–liposome complexes also did not alter both particle size and PDI, while the surface charge of both DNA–liposome complexes was decreased more significantly (*p* < 0.05) than that of the respective liposome. However, DNA–liposome complexes remained cationic.

**Table 1:** Particle size, polydispersity index (PDI), and zeta potential of different liposomes.

Formulation	Average size (nm)	PDI	Zeta potential (mV)
Dimethyldioctadecylammonium (DDA)	479 ± 87	0.31 ± 0.09	98 ± 3.6
DMT	408 ± 61	0.24 ± 0.06	77 ± 4.2^#^
pCMFO/DDA	495 ± 43	0.33 ± 0.09	73 ± 3.2^$^
pCMFO/DMT	417 ± 60	0.25 ± 0.07	62 ± 4.9*

Both DDA and DMT liposome were dispersed in Tris-buffer, respectively. pCMFO–liposome complexes were prepared by adding the plasmid pCMFO (50 µg/100 μL) into each liposome. Results are shown as mean ± SD from three batches. ^#^*p* < 0.05 compared with DDA, ^$^*p* < 0.05 compared with DDA, and *p < 0.05 compared with DMT.

**Figure 1:**
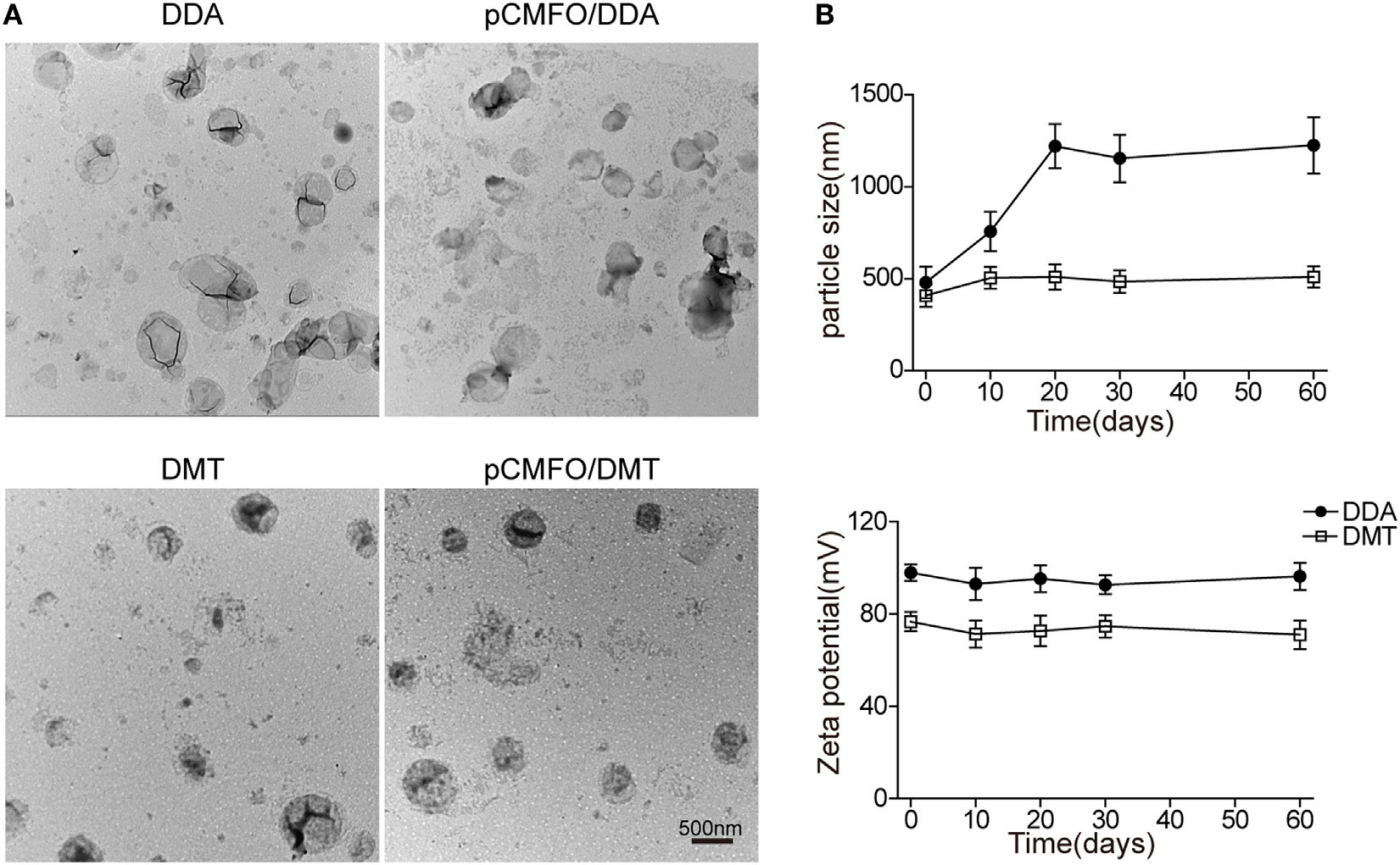
Characterization of both liposomes and pCMFO–liposome complexes. **(A)** TEM images of different liposomes including dimethyldioctadecylammonium (DDA), DMT, pCMFO/DDA, and pCMFO/DMT. **(B)** Comparison of particle size and zeta potential between DDA and DMT kept at 4°C for different days. Results are shown as mean ± SD of three batches.

As shown in Figure 1A, the DDA liposome was nearly spherical, although obvious signs of particle aggregates and some large vesicles could be observed. Addition of TDB and MPLA into the DDA liposome made the vesicles uniform spherical with little aggregation. Compared to each liposome alone, the plasmid DNA integrated into either the DMT liposome or the DDA liposome did not result in morphological change.

To evaluate the liposomal stability of DDA and DMT during storage at 4°C, both size and zeta potential were detected and compared with time. The DDA liposome was unstable because its particle size was increased rapidly during the first 20 days, while the DMT liposome remained constant for 2 months at 4°C. In addition, the zeta potential of both liposomes kept steady, although the DDA liposome had a higher value of zeta potential than the DMT liposome, during the whole experimental period (Figure 1B).

### Adsorption and Release of DNA *In Vitro*

**Figure 2:**
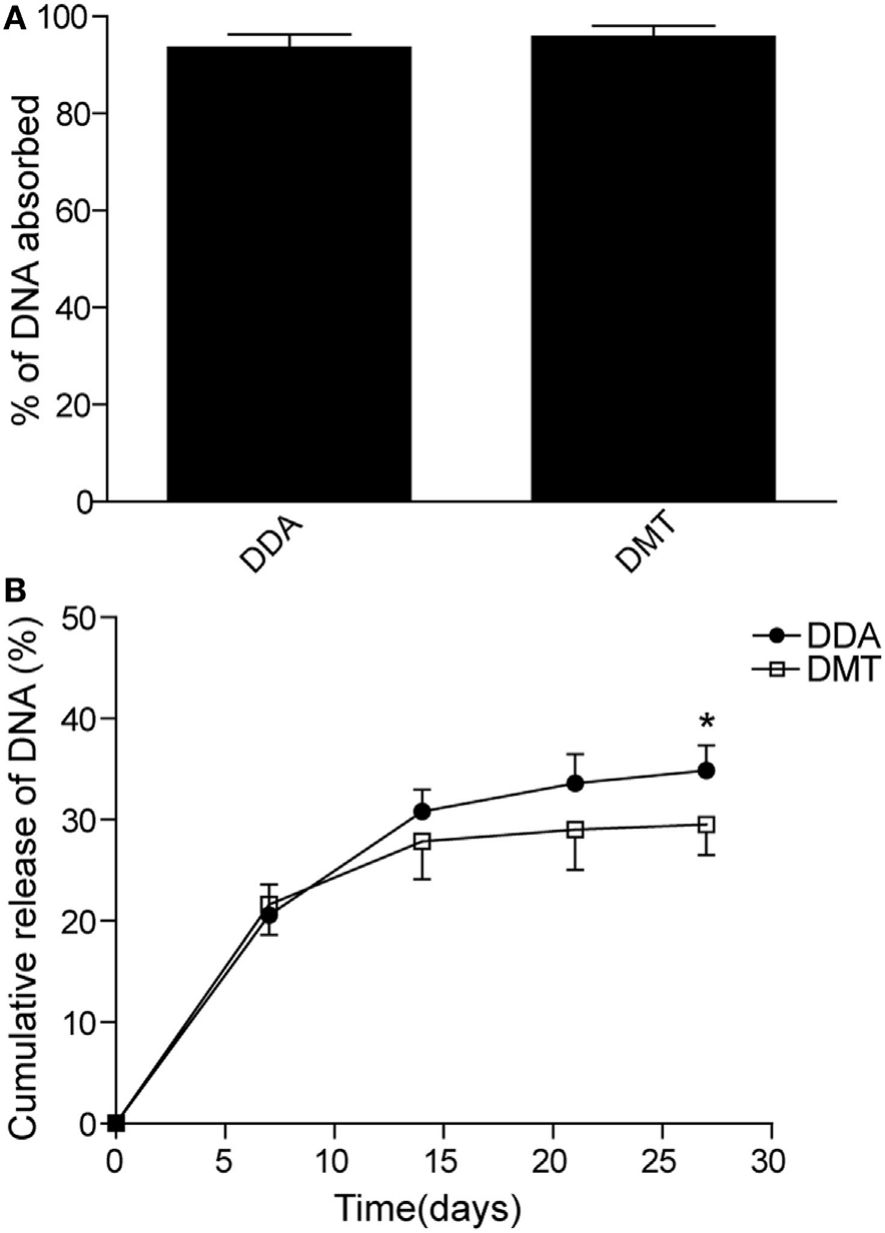
DNA adsorption and release rates controlled by different liposome–pCMFO complexes. **(A)** Comparison of the adsorption rate of the plasmid pCMFO to dimethyldioctadecylammonium (DDA) or DMT liposomes. **(B)** Comparison of the cumulative release rate of the plasmid pCMFO from liposome–DNA complexes at 37°C for 28 days. Results are shown as mean ± SD of three batches. *p < 0.05.

The adsorption of pCMFO (% of totally used) to both liposomes and the release of DNA (% of initially adsorbed) from pCMFO–liposome complexes were evaluated, respectively. Both DDA and DMT have equal ability to adsorb the plasmid DNA, and each liposome assimilated at least 95% of the total DNA used (Figure 2A). During the first 7 days after storage at 37°C, both DNA–DDA and DMT complexes released more than 20% of adsorbed DNA. At the 28th day after storage, the DNA–DDA complex resulted in approximately 35% cumulative loss of the adsorbed DNA, which was much higher than the DNA-DMT complex did (*p* < 0.05) (Figure 2B). Therefore, the DNA–DMT complex can release the plasmid pCMFO in a slower and longer-lasting way, when compared to the DNA–DDA complex.

### Enhanced and Longer-Lasting Protection Conferred by pCMFO/DMT Vaccine

**Figure 3:**
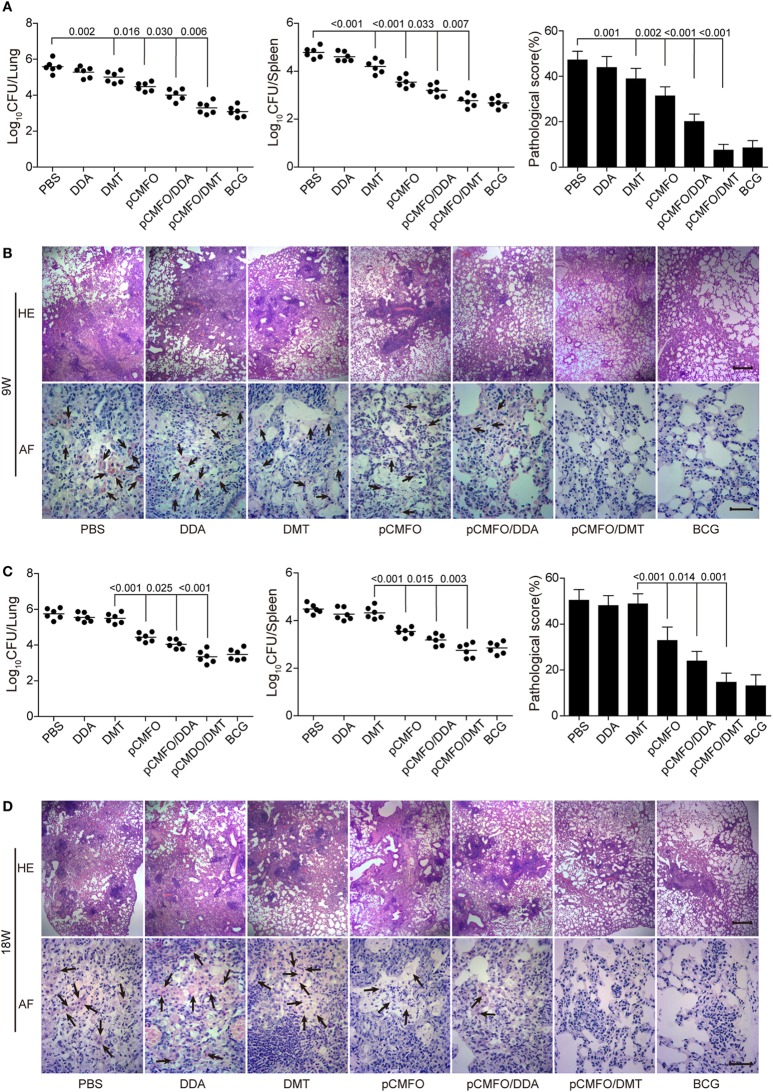
Comparison of the protective efficacy between different vaccinated mice against *M. tuberculosis* infection (*n* = 6). C57BL/6 mice were challenged with aerosol about 60 CFU of virulent *M. tuberculosis* H37Rv strain, 9 **(A,B)** and 18 **(C,D)** weeks after immunization. Four weeks post-challenge, the bacterial load in the lung and spleen of each mouse was enumerated. Results are shown as mean of log_10_ CFU ± SEM per organ of different groups. A *p*-value for the indicated comparisons is shown on the bar. Lung tissue sections were also prepared for hematoxylin-eosin (HE) staining (scale bar, 400 µm) and acid-fast (AF) staining (scale bar, 50 µm), respectively. Arrows indicate AF staining positive bacteria.

The short-term (Figures 3A,B) and long-term (Figures 3C,D) protection against aerosol infection with virulent *M. tuberculosis* H37Rv were compared and assessed in different vaccinated mice. Among all groups, PBS and DDA alone treated control mice had the highest bacterial load of *M. tuberculosis* in both lung and spleen, 9 and 18 weeks after immunization. Compared to the PBS control mice, DMT liposome alone only conferred non-specific protection at the ninth week (*p* < 0.05, Figure 3A). Stronger protection was observed in pCMFO vaccinated mice than control mice, regardless of time-points (*p* < 0.05, Figures 3A,C). Moreover, pCMFO/DDA also more significantly inhibited the growth of *M. tuberculosis* in the lung and spleen than the pCMFO group. Notably, the most significant inhibition of bacterial growth in both organs was obtained in the pCMFO/DMT group, which provided the comparable protection as BCG vaccine did, during the whole experimental period.

The results of lung tissue section stained with hematoxylin-eosin or acid-fast (AF) staining also supported the differential bacterial load in the lung of different groups. The most severe pathological lesion with granuloma-like formation in the lung were observed in the PBS and DDA control groups, with the highest pathological scores (Figures 3A,C) and AF positive bacteria pervaded the whole lung tissue section (Figures 3B,D). The pCMFO/DDA group had lesser lesions in the lung and much lower pathological scores than pCMFO and DMT vaccinated mice, respectively. A few AF positive bacilli were persistently located in the lung of these three groups (Figures 3B,D). Importantly, both pCMFO/DMT and BCG vaccinated mice had the fewest lesions and the lowest scores of all groups, with few AF positive bacilli observed in the lung section during the whole experimental period.

### Th1-Type Biased Responses to Each Subcomponent Induced by pCMFO/DMT

**Figure 4:**
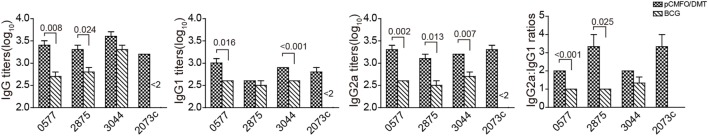
The protein CMFO subcomponent-specific antibodies at the ninth week after immunization (*n* = 6). Mice were immunized with pCMFO/DMT, Bacillus Calmette–Guérin (BCG) and PBS, respectively. 9 weeks later, Rv0577, Rv2875, Rv3044, and Rv2073c-specific antibodies, including IgG, IgG1, and IgG2a in the serum of each mouse were tittered by ELISA. Results are shown as mean (±SEM) log10 endpoint titer and the ratio of IgG2a/IgG1 in vaccinated mice (*n* = 6). A *p* value for the indicated comparison is shown on the bar.

In order to assess the contribution of each subcomponent consisted of the fusion protein CMFO, mice were immunized with PBS, pCMFO/DMT and BCG, respectively. 9 weeks later, Rv0577, Rv2875, Rv3044, and Rv2073c-specific antibodies, including IgG, IgG1, and IgG2a in the serum of each mouse were tittered by ELISA (Figure 4). BCG vaccinated mice did not produce any antibodies against the antigen Rv2073c as expected. pCMFO/DMT vaccinated mice produced much higher titers of IgG, IgG1, and IgG2a against each antigen than the BCG group, except anti-Rv3044 IgG and anti-Rv2875 IgG1 antibodies (*p* < 0.05, Figure 4). Correspondingly, the ratio of IgG2a/IgG1 response to each subcomponent in the pCMFO/DMT group was higher than the BCG group.

**Figure 5:**
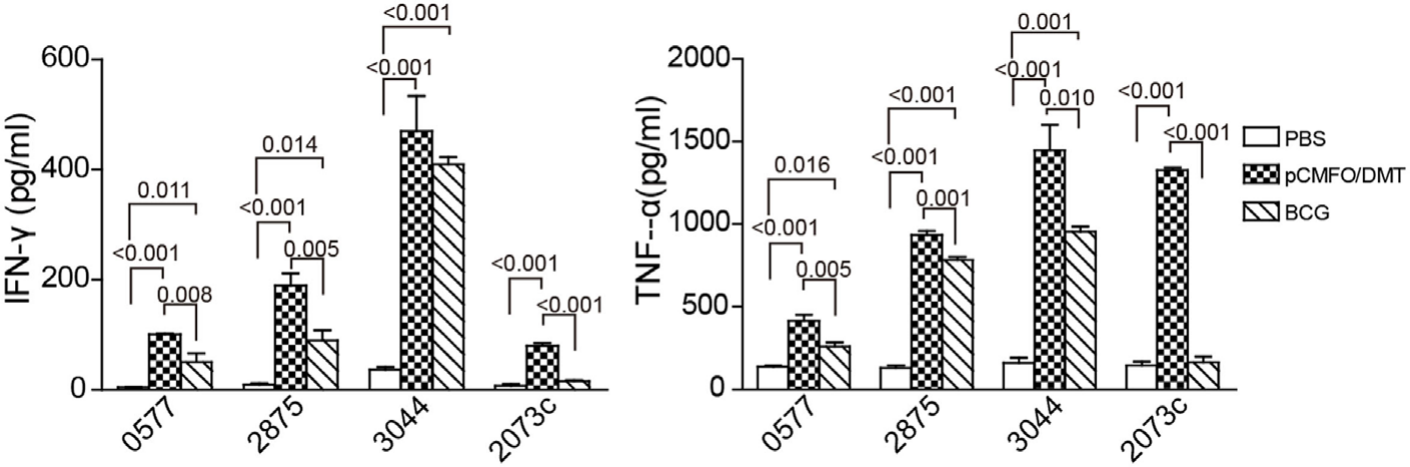
The protein CMFO subcomponent-specific cytokines at the ninth week after immunization (*n* = 6). Mice were immunized with pCMFO/DMT, Bacillus Calmette–Guérin (BCG), and PBS, respectively. 9 weeks later, 5 × 10^6^ splenocytes from each mouse in different groups were incubated with Rv0577, Rv2875, Rv3044, or Rv2073c, respectively. RPMI1640 medium was used as negative control. 24 h later, supernatants were collected and the concentration of Th1 (TNF-α and IFN-γ), Th2 (IL-4), and Th17 (IL-17A) was analyzed by a cytometric bead array kit. The concentration of antigen-specific cytokines was calculated by subtracting the value of the medium control. Results are expressed as mean ± SD (pg/mL). A *p*-value for the comparison among different groups is shown above the bars in panels. There was almost no production of IL-4, and IL-17A in all groups and data were not shown.

Moreover, subcomponent-specific cytokines including Th1, Th2, and Th17 in the supernatant of splenocytes were detected by a CBA kit. As expected, the BCG group, as well as the PBS group, did not induce any Rv2073c-specific cytokines (Figure 5). Splenocytes from both pCMFO/DMT and BCG groups secreted much higher levels of TNF-α and IFN-γ than that of control mice, when incubated with antigens Rv0577, Rv2875, and Rv3044 *in vitro*, respectively. Importantly, pCMFO/DMT elicited higher levels of TNF-α response to four subcomponents alone than BCG vaccine. Among all subcomponents, Rv3044 had the strongest immunogenicity with the highest levels of both INF-γ and TNF-α secreted by splenocytes from pCMFO/DMT vaccinated mice. There was no statistic difference of INF-γ response to Rv3044 between BCG vaccination and the pCMFO/DMT group. However, the level of INF-γ response to other subcomponents in the BCG group was lower than that of pCMFO/DMT vaccinated mice. The concentrations of IL-4 and IL-17A in the supernatant from all groups were extremely low, less than 1 pg/mL.

### Stronger Th1-Type Responses Induced by pCMFO/DMT than pCMFO/DDA

**Figure 6:**
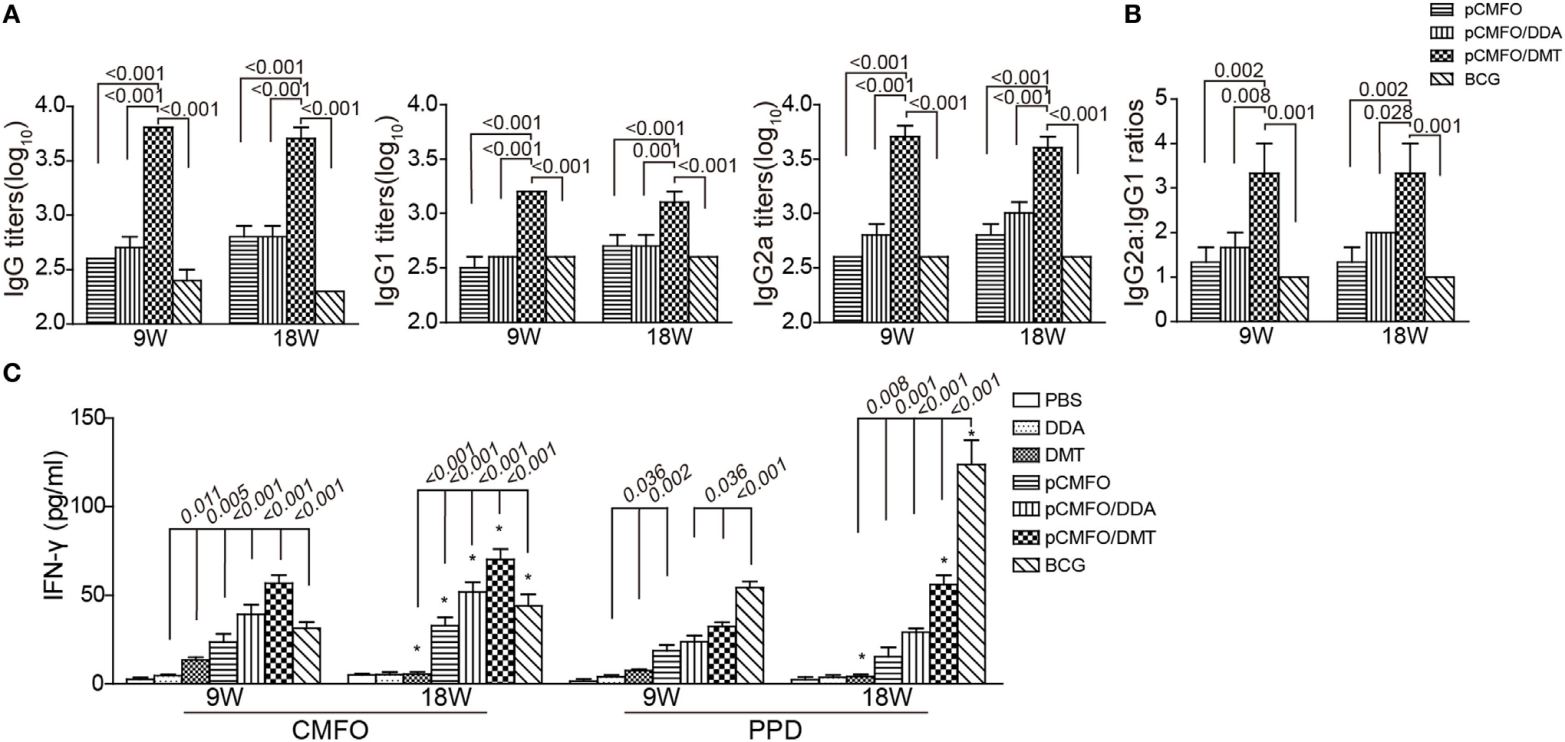
Th1-type biased immune responses in immunized mice at the ninth and 18th week after immunization (*n* = 6). Mice were immunized with pCMFO, pCMFO/DDA, and pCMFO/DMT, respectively. Bacillus Calmette–Guérin (BCG) was used as a positive control, and PBS, dimethyldioctadecylammonium (DDA), and DMT alone were performed as negative controls. **(A)** IgG, IgG1, and IgG2a antibodies against CMFO in sera were detected by ELISA. Results are shown as mean (±SEM) log10 endpoint titer. **(B)** The ratio of IgG2a/IgG1 in the different vaccinated mice. **(C)** The levels of PPD or CMFO-specific IFN-γ secreted by splenocytes. 5 × 10^6^ of splenocytes from each mouse in different groups were re-stimulated with 10 µg/mL of CMFO or PPD for 72 h at 37°C, 5% CO_2_. The supernatants were collected and ELISA was used to detect the concentration of IFN-γ. Results are expressed as mean ± SD (pg/mL). A *p*-value for the comparison between different groups is shown above the bars in panels. *p < 0.05, 9 vs. 18 weeks of the same vaccinated mice.

To compare the immunogenicity of pCMFO–liposome complexes, CMFO-specific antibodies, including IgG, IgG1, and IgG2a in the sera of different vaccinated mice were tittered by ELISA, 9 and 18 weeks after vaccination (Figure 6A). No CMFO-specific antibodies were measured in DDA, or DMT treated control mice (data not shown). Compared with control mice, pCMFO-based vaccines, as well as the BCG group, induced higher levels of antibodies against the antigen CMFO. The levels of antibodies in these groups kept steady at two time-points. Importantly, the highest levels of CMFO-specific IgG, IgG1, and IgG2a antibodies were elicited in pCMFO/DMT vaccinated mice of all groups, during the whole experimental period (*p* < 0.05). In addition, the highest ratio of IgG2a/IgG1 was also obtained in the pCMFO/DMT group, among all groups (*p* < 0.05, Figure 6B).

As shown in Figure 6C, PBS and DDA alone treated mice showed the lowest levels of PPD or CMFO-specific IFN-γ of all groups. Splenocytes from the DMT alone vaccinated mice also secreted more quantity of IFN-γ than both PBS and DDA treated mice at the ninth week. Compared to control mice, higher levels of CMFO-specific IFN-γ were elicited in pCMFO, pCMFO/DDA, pCMFO/DMT, and BCG vaccinated mice (*p* < 0.05), which also increased over time. In particular, the pCMFO/DMT group, of all groups, conferred the strongest IFN-γ response to the antigen CMFO at both time-points, whereas the highest level of PPD-specific IFN-γ was induced by BCG vaccination (*p* < 0.05). Taken together, Th1-type immune responses might be more easily induced in the pCMFO/DMT group, compared with pCMFO, pCMFO/DDA, and BCG vaccinated mice.

### Higher Levels of Memory T Cells Induced by pCMFO/DMT than pCMFO/DDA

**Figure 7:**
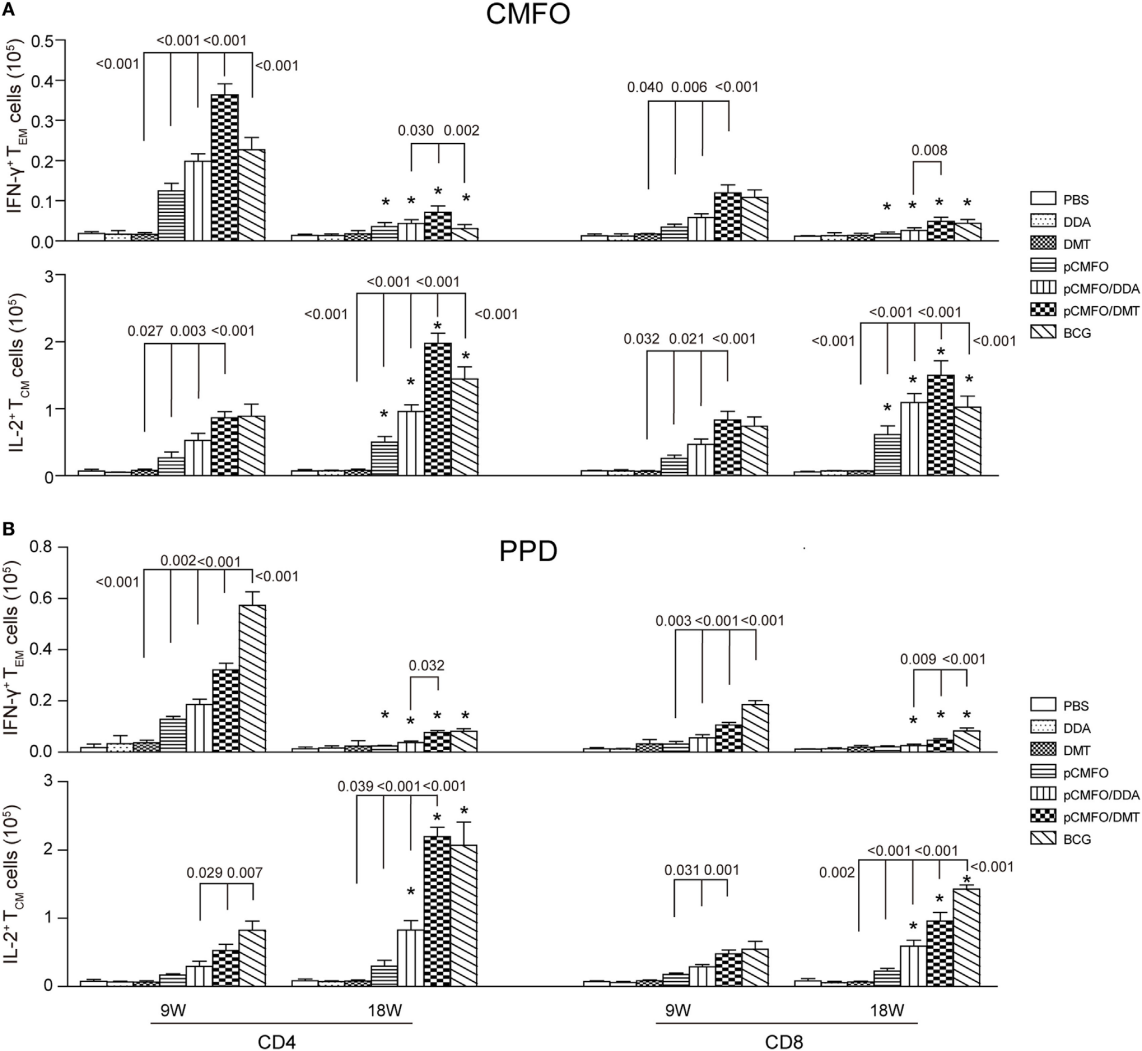
Antigen-specific memory T cells in the spleen of different vaccinated mice (*n* = 6). 9 and 18 weeks after immunization, splenocytes were prepared from each mouse immunized with pCMFO, pCMFO/DDA, and pCMFO/DMT, respectively. Bacillus Calmette-Guérin (BCG) was used as a positive control. PBS, dimethyldioctadecylammonium (DDA), and DMT alone were performed as negative controls. The subsets of CMFO- or PPD-specific CD4^+^ or CD8^+^ effector and central memory T cells (T_EM_ and T_CM_) were identified by intracellular cytokines staining and a multicolor flow cytometer. The gate strategy was shown in Figure S2 in Supplementary Material. **(A)** The absolute number of CFMO-specific IFN-γ secreting T_EM_ cells and IL-2 secreting T_CM_ cells. **(B)** The absolute number of PPD-specific IFN-γ secreting T_EM_ cells and IL-2 secreting T_CM_ cells. The results are expressed as mean ± SD. A *p*-value for the comparison between different groups is shown above the bars in panels. *p < 0.05, 9 vs. 18 weeks of the same vaccinated mice.

Regardless of 9 and 18 weeks after immunization, PBS, DDA, and DMT treated control mice only induced the lowest levels of PPD- or CMFO-specific IFN-γ^+^ T_EM_ cells or IL-2^+^ T_CM_ cells in the spleen of all groups (*p* < 0.05, Figure 7). pCMFO/DDA vaccinated mice not only produced much higher numbers of CMFO-specific IFN-γ^+^ CD4^+^ and CD8^+^T_EM_ cells than pCMFO alone at the ninth week, but also elicited more IL-2^+^ T_CM_ cells at two time-points. More importantly, higher levels of PPD or CMFO-specific IFN-γ^+^ T_EM_ cells or IL-2^+^ T_CM_ cells elicited by pCMFO/DMT vaccinated mice than both pCMFO/DDA and pCMFO groups during the whole experimental period (*p* < 0.05). In particular, PPD- or CMFO-specific IL-2^+^ CD4^+^ and CD8^+^ T_CM_ cells dominated in the pCMFO/DMT vaccinated mice, which increased over time.

Although there was no statistic difference of CMFO-specific IL-2^+^ CD4^+^ or CD8^+^T_CM_ cells between the pCMFO/DMT and BCG vaccinated mice at the ninth week, pCMFO/DMT vaccinated mice elicited many more CFMO-specific IL-2^+^ T_CM_ cells than BCG group after 18 weeks (*p* < 0.05, Figure 7A). In contrast, BCG vaccination induced the highest levels of PPD-specific T_EM_ and T_CM_ cells, among all groups (*p* < 0.05, Figure 7B). PPD-specific IL-2^+^ CD4^+^ T_CM_ cells were induced more significantly in the BCG group than the pCMFO/DMT group at the ninth week, but raised to the same level in both groups at 18th week.

## Discussion

Considering the serious threat of TB to the public health, the development of new generation vaccine for TB remains a global priority. Improving the immunogenicity of DNA vaccine would benefit its clinical use. As a delivery vehicle, the DDA liposome could mediate the initial contact with the surface of antigen-presenting cells (APCs) (33). The antigen carried by the liposome would be released slowly from the antigen–liposome complex and is apt to deposit at the site of vaccination (34). TDB alone could activate APCs through a mincle receptor (35) and FcRgamma-Syk-Card9 pathway (36), which produces MyD88-dependent Th1 and Th17 responses (37). MPLA is a typical agonist of toll-like receptor 4 (38) and could enhance CD4^+^ T cell responses (39) and promote the maturation of dendritic cells through TRIF and MyD88 signaling pathways (40). The DDA liposome-based adjuvants have been widely studied in different subunit protein vaccines (33–35, 41, 42). Previous studies reported that DDA alone adjuvanted vaccine is safe, which could induce antibody, weak Th1 and Th17 responses to antigens (43). Compared to DDA alone, DDA/MPLA is a stronger inducer of antigen-specific IFN-γ and IL-17 responses (43). Combinations of DDA and TDB (adjuvant CAF01) with BCG could induce more CD4^+^ and CD8^+^ T cells expressing IFN-γ and IFN-γ/TNF-α in vaccinated mice than BCG alone (44). CAF01 or DDA/MPLA adjuvanted different subunits vaccinated mice were demonstrated to elicit mainly polyfunctional CD4^+^ T cells secreting IFN-γ, IL-2, and TNF-α, and TNF-α^+^IL-2^+^ CD4^+^ T cells responses (43, 45). Although CD4^+^ Th1 responses have been accepted to play a critical role in the vaccine-induced protection against TB infection, the levels of IFN-γ^+^IL-2^+^ CD4^+^ T cells were related with the better protection induced by a subunit vaccine with the mixture of TDB and MPLA formulated into oil-in-water (adjuvant MTO) (21). When compared with the MTO, MTOM (a mixture of an addition of heat-killing *Mycobacterium vaccae* into the MTO) adjuvanted protein vaccine could enhance the levels of antigen-specific single and multifunctional IL-2^+^ T cells more significantly and confer the comparable protection with the BCG vaccine against the infection of *M. tuberculosis* in mice (32). Because of complexity of the adjuvant MTOM, the DMT liposome was established as a novel adjuvant of subunit protein vaccine and is a strong inducer of Th1-biased responses and IFN-γ^+^IL-2^+^ T cells, especially IL-2^+^CD4^+^ T_CM_ cells (20, 22, 30). However, the action mechanism and the adjuvant effect of the DMT liposome on DNA vaccine remain to be investigated.

In this study, TDB and MPLA added into the DDA liposome decreased the surface charge of the liposome. A previous study reported that the integration of TDB into the DDA liposome had no obvious effects on the surface charge (41). Because MPLA has a negatively charged phosphate group, the addition of MPLA may reduce the surface charge of the DMT liposome by electrostatic interaction, as confirmed in this study. Since the plasmid DNA also possesses a negatively charged phosphate group, the electrostatic interaction between DNA and cationic liposome may lead to a further drop of the surface charge of DNA-liposome complexes, which might have a significant effect on the stability of DMT adjuvanted DNA vaccine. As demonstrated in this study, the incorporation of TDB and MPLA into the DDA liposome enhanced the storage stability at 4°C for at least 2 months, which was consistent with a previous report (42). Importantly, the preparation has no significant influence on the adsorption of the plasmid DNA to the DMT liposome but prolongs the retention time of the plasmid DNA *in vitro*, when compared to the DDA liposome. Therefore, the plasmid DNA, and two receptor agonists TDB and MPLA might be released simultaneously and slowly from the pCMFO/DMT complex and deposited at the injection site after immunization. Correspondingly, pCMFO/DMT induced Th1-biased immune responses and a stronger protection than pCMFO/DDA, while both DDA and DMT liposomes could enhance the immunogenicity and protection of the pCMFO DNA vaccine against *M. tuberculosis* infection in vaccinated mice. The controlled release effect of the DMT liposome on the plasmid DNA and the agonists might attribute to the augmented immunogenicity and enhanced protection.

Dimethyldioctadecylammonium liposome alone, or the integration of TDB or MPLA into the DDA liposome may result in early responses of both IL-17 and IFN-γ in vaccinated mice (43, 46). In this study, splenocytes from pCMFO/DMT vaccinated mice did not secrete any subcomponent-specific IL-17A at the ninth week, although we did not detect earlier and longer-lasting IL-17A responses after immunization. In fact, a clinical trial of CAF01 adjuvanted Ag85B-ESAT6 subunit vaccine also demonstrated that only low level of antigen-specific IL-17 (less than 20 pg/mL) was detected in immunized persons, 14 and 32 weeks after immunization (47). IL-17 is involved in an immune protection against *M. tuberculosis* through recruiting and activating neutrophils at an early stage of infection, over-stimulation of Th17 responses has instead associated with TB pathology and progression (48).

Memory T cells are newly discovered to be an effective biomarker to assess vaccine-induced protection against TB (20, 22, 49). When encountered a specific antigen, T_EM_ cells can secrete IFN-γ, activating macrophages to kill the intracellular *M. tuberculosis*. T_CM_ cells express IL-2, which can promote the proliferation of memory T cells and then differentiate into effector T cells rapidly (50). Mice vaccinated with pCMFO/DMT elicited higher levels of IFN-γ^+^ T_EM_ cells and IL2^+^ T_CM_ cells than pCMFO/DDA group and IL2^+^ T_CM_ cells were dominated by the pCMFO/DMT group. Recombinant live vaccine rBCG VPM1002 being conducted in a clinical trial could provide more significant protection against *M. tuberculosis* infection than the parent BCG, and increased level of T_CM_ cells in VPM1002 vaccinated mice was associated with the enhanced protection (51). In addition, a cocktail of rBCG strains ABX also stimulated higher numbers of IL-2^+^CD8^+^ T_CM_ cells in mice (49). As attenuated live vaccines, BCG and these recombinant BCG strains could replicate for a short time in the vaccinated host (52). 239 antigens can be persistently secreted by live BCG vaccine (53). Therefore, almost no antigen-based vaccines could provide better protection against primary TB infection than the BCG vaccine. Slow process and persistent release of the plasmid DNA, TDB, and MPLA from the pCMFO/DMT complex might have a similar function as attenuated live vaccines. pCMFO/DMT vaccinated mice produced higher levels of each subcomponent-specific antibodies and multiple cytokines, compared with BCG and PBS groups. In fact, one single antigen, e.g., Rv3044, may induce more significant immune responses than others following pCMFO/DMT immunization. As expected, each single antigen of the fusion protein CMFO only provided inferior protection to the BCG vaccine. However, pCMFO/DMT could confer the comparable protection efficacy against primary infection as BCG vaccine did. Both multistage vaccines CAF01 adjuvanted Ag85B-ESAT6-Rv2660c (45) and CMFO in adjuvant of DMT (22) also support our current results. Importantly, these vaccines also showed the effects to inhibit the growth of latent *M. tuberculosis* in different animal models (22, 45, 54). In this study, BCG could not induce higher levels of Th1-typed immune responses to latency-related antigens, which may attribute to the inferior effects of BCG vaccine to thwart the reactivation of latent TB infection in animals, as demonstrated in previous studies (22). Because of safety, pCMFO/DMT could replace live BCG vaccine as prophylaxis in immunocompromised individuals. The effects of pCMFO/DMT on the prevention of latent TB infection remain to be investigated.

In conclusion, the delivery and controlled release effect of the DMT liposome might be related to the enhanced efficacy of DMT adjuvanted vaccines against TB, and pCMFO/DMT is a very promising vaccine for the protection against primary TB infection. Our findings lay the foundation for further evaluation of DMT adjuvanted vaccines in preclinical and clinical trials.

## Ethics Statement

Animal experiments were performed in accordance with the guidelines of the Chinese Council on Animal Care. The research protocols were approved by the Committee on the Ethics of Animal Experiments of Tongji Medical College, Huazhong University of Science and Technology.

## Author Contributions

This project was designed by XF. MT, ZZ, ST and LL performed the experiments. MT and XF analyzed the data. MT wrote the manuscript. The final manuscript was revised thoroughly by XF. NU participated in the experiments for the revised manuscript.

## Conflict of Interest Statement

DMT-adjuvanted pCMFO DNA vaccine was applied for the invention patent of China (no. CN 201810124108.X).

## Supplementary Material

Figure S1 Construction and identification of the recombinant eukaryotic expression plasmid pCMFO. **(A)** The structural diagram of the recombinant eukaryotic expression plasmid pCMFO. In order to facilitate the secretion of the protein CMFO from transfected cells, the recombinant plasmid pCMFO was constructed successfully by subcloning the fusion gene encoding the SP_tPA_ and the protein CMFO into the eukaryotic expression vector pVAX1. **(B)** The expression of the protein CMFO from the plasmid pCMFO-transfected HEK 293T cells was confirmed by western blotting with anti-CMFO mouse sera. The protein CMFO with an expected molecular mass of about 128 kDa was only present in the supernatant, rather than in the lysate from the pCMFO-transfected HEK 293T cells. As expected, the control vector pVAX1-transfected cells did not express the protein CMFO. Therefore, the protein CMFO can be expressed and secreted from the pCMFO-transfected cells under the direction of both CMV promoter and SP_tPA_.

Figure S2 The gating strategy of the identification of memory T cells in the splenocytes from different vaccinated mice by intracellular flow cytometry analysis.
