# Supplementary figures and images for: Formulation in DDA-MPLA-TDB Liposome Enhances the Immunogenicity and Protective Efficacy of a DNA Vaccine against Mycobacterium tuberculosis Infection
Source: Front Immunol. 2018 Feb 27;9:310. doi: 10.3389/fimmu.2018.00310 (PMC5835323; doi:10.3389/fimmu.2018.00310)

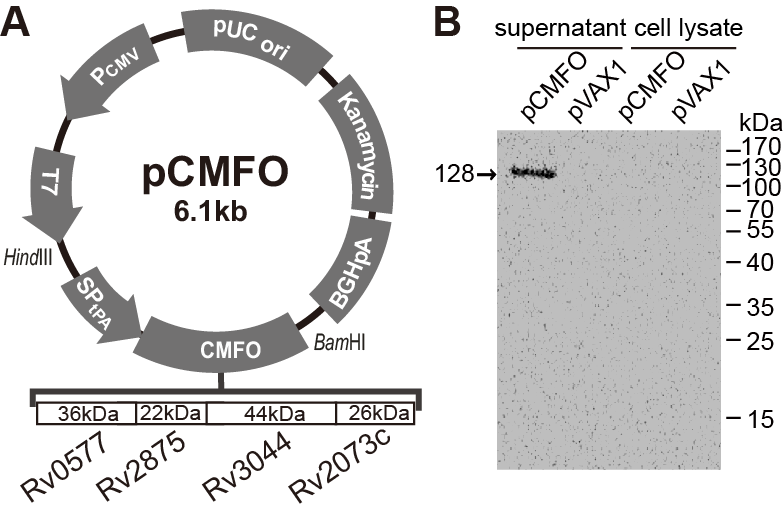

Supplement: Figure S1 — Construction and identification of the recombinant eukaryotic expression plasmid pCMFO. (A) The structural diagram of the recombinant eukaryotic expression plasmid pCMFO. In order to facilitate the secretion of the protein CMFO from transfected cells, the recombinant plasmid pCMFO was constructed successfully by subcloning the fusion gene encoding the SPtPA and the protein CMFO into the eukaryotic expression vector pVAX1. (B) The expression of the protein CMFO from the plasmid pCMFO-transfected HEK 293T cells was confirmed by western blotting with anti-CMFO mouse sera. The protein CMFO with an expected molecular mass of about 128 kDa was only present in the supernatant, rather than in the lysate from the pCMFO-transfected HEK 293T cells. As expected, the control vector pVAX1-transfected cells did not express the protein CMFO. Therefore, the protein CMFO can be expressed and secreted from the pCMFO-transfected cells under the direction of both CMV promoter and SPtPA. [file image_1.tif]

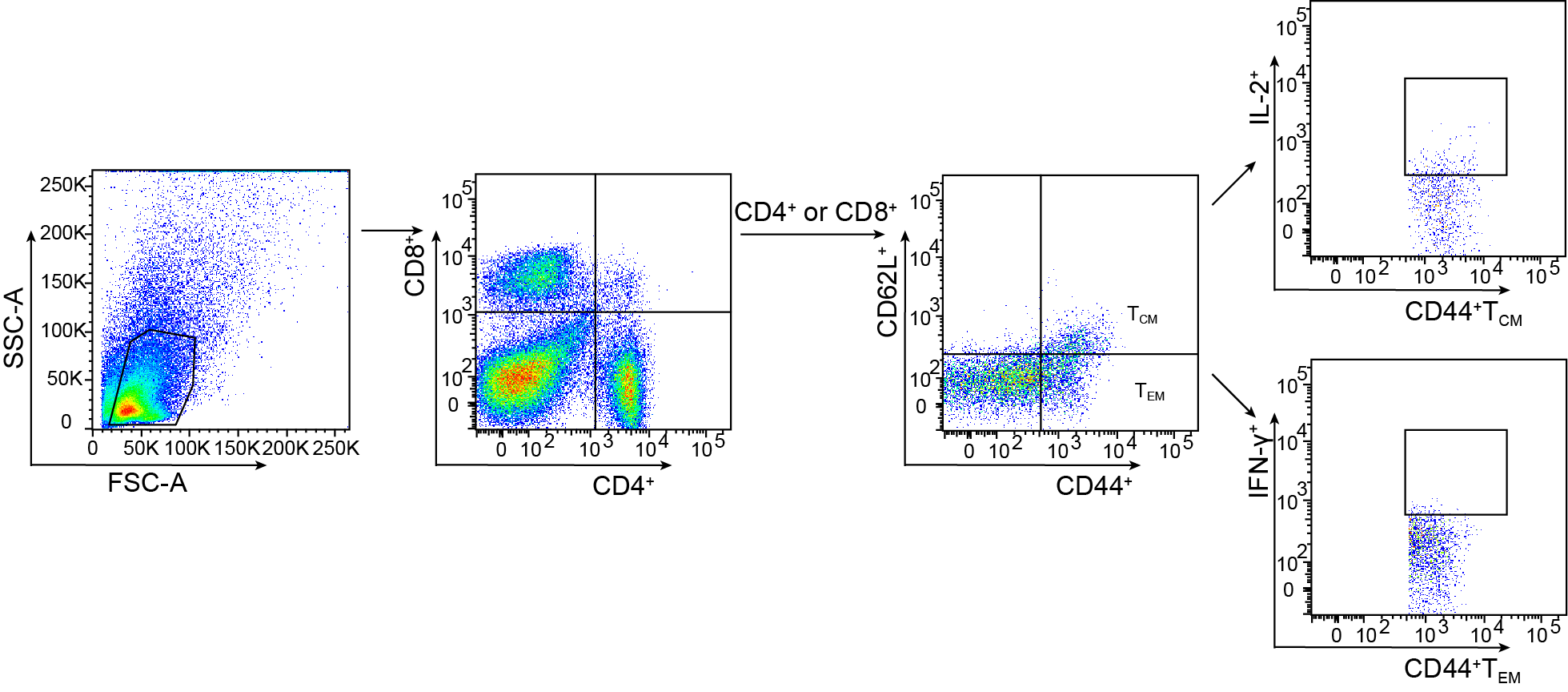

Supplement: Figure S2 — The gating strategy of the identification of memory T cells in the splenocytes from different vaccinated mice by intracellular flow cytometry analysis. [file image_2.tif]
